# Fatal Human Case of Western Equine Encephalitis, Uruguay

**DOI:** 10.3201/eid1705.101068

**Published:** 2011-05

**Authors:** Adriana Delfraro, Analía Burgueño, Noelia Morel, Gabriel González, Alicia García, Juan Morelli, Walter Pérez, Héctor Chiparelli, Juan Arbiza

**Affiliations:** Author affiliations: Universidad de la República, Montevideo, Uruguay (A. Delfraro, A. Burgueño, J. Arbiza);; Ministerio de Salud Pública, Montevideo (N. Morel, H. Chiparelli);; Hospital Británico, Montevideo (G. González, A. Garcia, J. Morelli, W. Pérez)

**Keywords:** encephalomyelitis, Western equine encephalitis virus, viruses, humans, Uruguay, letter

**To the Editor:** The genus *Alphavirus* (family *Togaviridae*) comprises 29 viral species (*1*), grouped in at least 7 antigenic complexes by their serologic cross-reactivity (*2*). They are maintained in nature through enzootic cycles involving arthropods as vectors with subsequent amplification in small mammals or birds, and epizootic cycles between mosquitoes and large mammals such as horses or humans.

Few reports have been made of the circulation of alphaviruses in Uruguay. A serologic study conducted in 1970 found antibodies to western (WEEV) and eastern equine encephalitis viruses by using complement fixation and hemagglutination inhibition tests in serum specimens from children (*3*). In 1972–1973, epizootics in horses caused by WEEV were reported in Argentina and Uruguay, and WEEV was isolated from a sick horse (*4*).

We report a fatal case of viral encephalitis in April 2009 in Montevideo, Uruguay, in a previously healthy 14-year-old boy. Four days before he sought treatment, he had fever, asthenia, and headaches. At hospital admission (April 12, 2009), he was febrile and without neurologic signs; amoxicillin treatment was initiated. Results of a computed tomography scan of the brain were normal.

On day 1, headache, vomiting, neck stiffness, and partial left seizures on the left side developed. Also exhibited were consciousness depression (Glasgow Coma Scale 12 points), hyperreflexia, and bilateral Babinski sign. A cerebrospinal fluid (CSF) sample was negative for bacteria in cultures. An electroencephalogram showed diffuse brain suffering. The patient was brought to the intensive care unit with a clinical diagnosis of viral encephalitis. Over the next 24–36 hours, intracranial hypertension developed, and medical treatment was given (sedation, hyperventilation, mannitol, and barbiturates). Conscience depression progressed to a deeper level, and a computed tomography scan of the brain showed dilatation of the temporal ventricles and compression of the peritroncal and sylvian cisterns. During the next 48 hours, the coma level went deeper, reaching 6 on the Glasgow Scale. Another CSF specimen was taken, and PCR results were negative for herpesvirus and enterovirus. Glasgow Coma Scale level was 3 on April 15, and a decompressive craniectomy was done. Seventy-two hours after admission, the patient died.

The patient’s plasma and CSF were tested for antibodies to dengue and West Nile viruses (immunoglobulins M and G) through ELISA (Focus Technologies, Cypress, CA, USA) and for St. Louis encephalitis and dengue virus by M antibody-capture–ELISA (*5*). RNA was extracted from plasma and CSF, followed by a generic nested reverse transcription–PCR (RT-PCR) for flaviviruses (*6*). Serologic and molecular test results were negative for the above-mentioned pathogens. Then we performed a generic nested RT-PCR (*7*), which amplifies 448 bp at first round and 195 bp (second round) of the alphavirus NSP4 gene. Also, a heminested PCR was done (products 372 bp and 303 bp); RNA from Venezuelan equine encephalitis virus Tc-83 (provided by M. Contigiani, Universidad de Córdoba) was used as positive control. The target region is informative enough to allow the precise identification of the most relevant alphaviruses by sequencing and phylogenetic analysis. Alphavirus genome amplification was achieved for the CSF specimen collected at admission to the hospital. Plasma and a second CSF specimen were PCR negative. To confirm these findings, another nested RT-PCR reaction targeting the NSP1 gene was done as described previously (*8*). A 208-bp amplicon, which corresponded to the expected size for WEEV, was obtained from plasma and the first CSF specimen.

Sequence analyses were conducted on the NSP4 fragments. Maximum likelihood (*9*) and Bayesian (*10*) phylogenetic analyses gave similar results. The Figure, panel A, shows that sample LCR/09-303 is part of a well-supported clade (aLRT = 0.99), which groups WEEVs. The sequence LCR/09-303 is a sister taxon to sequences GQ287641 and GQ287642, with poor support (Figure, panel B) (Table A1). These are reference WEEV USA strains (Imperial and Kern) obtained from *Culex tarsalis* mosquitoes. Our sample and the mentioned sequences are part of a well-supported clade (aLRT = 0.85), together with GQ287645, AF214040, and FJ786260. These are also USA strains; 2 were isolated from infected horses and 1 from *Cx. tarsalis* mosquitoes. Notably, our sequence is distantly related to GQ287646, which was isolated from *Culex* spp. mosquitoes in Chaco, Argentina. The nucleotide sequence of the positive control VEEV-Tc83 is correctly placed in the VEEV clade.

**Figure:**
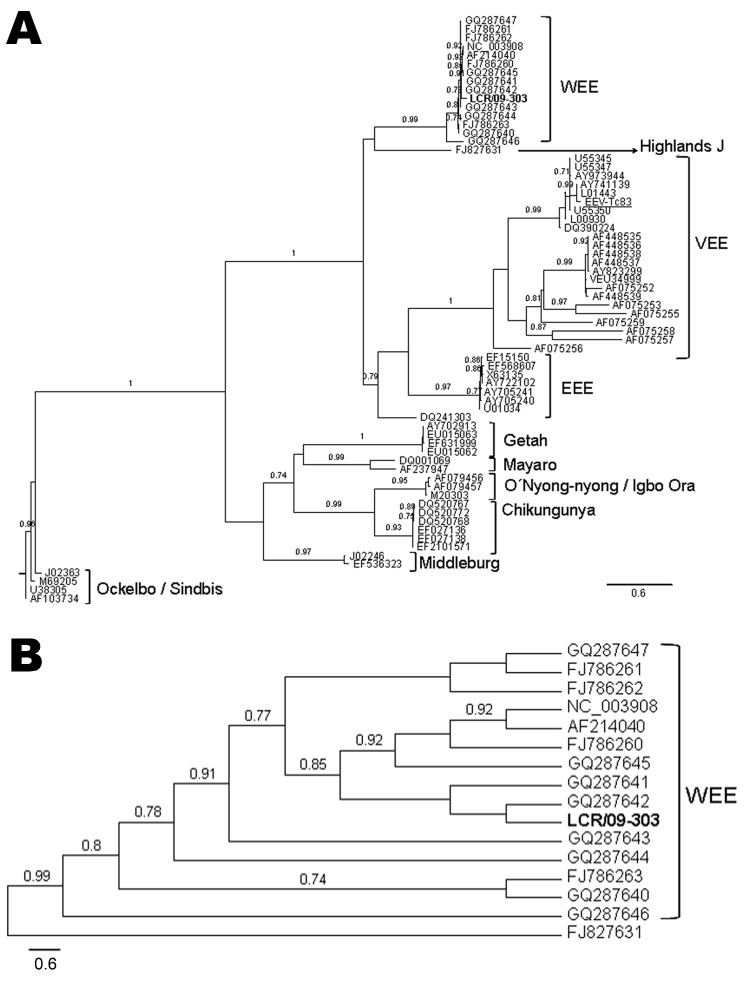
A) Phylogenetic tree obtained by maximum likelihood analysis of sequences corresponding to the alphavirus NSP4 gene. Alignment used in the analysis had 448 bp and was conducted by using BioEdit software version 7.0.9.0 (www.mbio.ncsu.edu/BioEdit/BioEdit.html). Estimation of the suitable model of nucleotide substitution was carried out by using Modelgenerator (http://bioinf.may.ie/software/modelgenerator). Phylogenetic analysis was run on the PhyML web server (www.atgc-montpellier.fr/phyml), with the following settings: nucleotide substitution model: general time reversible + proportion invariant + Γ; proportion of invariable sites: 0.39; gamma distribution parameter α: 0.67; node support: approximate likelihood-ratio test (only values over 0.70 are shown). Sequences included in the analysis were the following (GenBank accession numbers for individual isolates provided when applicable): human encephalitis case-patient: LCR/09-303 (**boldface**); reverse transcription–PCR positive control Venezuelan equine encephalitis virus [VEEV] Tc83 (282 nt), FJ786261; western equine encephalitis virus (WEEV): AF214040, GQ287647, GQ287646, GQ287645, GQ287644, GQ287643, GQ287642, GQ287641, GQ287640, FJ786263, FJ786262, FJ786260, NC003908; Highlands J virus, FJ827831; Venezuelan equine encephalitis virus (VEEV), L01443, DQ390224, AF075255, AY823299, AF448539, AF448538, AF448537, AF448536, AF448535, AF075252, U34999, AF075259, AF075256, AF075253, AF075257, AF075258, AY973944. L00930, AY741139, U55350, U55347, U55345; eastern equine encephalitis virus (EEEV), AY722102, U01034, EF568607, EF15150, AY705241, AY705240, X63135, DQ241303; Getah virus, EU015063, EU015062, EF631999, AY702913; Mayaro virus, AF237947, DQ001069; M20303; O’nyong-nyong virus, AF079456; Igbo Ora virus, AF079457; chikungunya virus, EF210157, EF027138, EF027136, DQ520772, DQ520768, DQ520767; Middleburg virus, EF536323, J02246; Ockelbo virus, M69205: Sindbis virus, AF103734, U38305, J02363, M69205. B) Detail of the WEEV clade, showing the relationships between the sample LCR/09-303 and the rest of the WEEV isolates included in the analysis. Scale bars indicate expected nucleotide changes per site.

Clinical and laboratory findings showed that the illness described here was compatible with viral encephalitis. Using a generic RT-PCR assay on an early CSF sample, we amplified a partial sequence (NSP4 gene) of an alphavirus. Phylogenetic analyses showed that the patient’s sequence grouped with sequences from WEEV, with high statistical support. A second RT-PCR assay on the NSP1 gene enabled us to obtain an amplification of 208 bp, which is consistent with the expected size for WEEV. Therefore, we concluded that the fatal disease was likely caused by WEEV. Since the 1970s, to our knowledge, the presence of WEEV (or other alphaviruses) in Uruguay has not been documented. Moreover, no recent reports have been made of genome detection of WEEV in encephalitis cases in the region.

Although the case described here may be rare, the etiology of many viral encephalitides in Uruguay remains unknown. Serologic studies in horses and studies to detect arboviruses in mosquito populations are being conducted to investigate the status of arbovirus infections in Uruguay.

## Appendix

**Table A1:** Strain designation, place and date of isolation, and encephalitis virus species

Strain designation	Place of isolation	Year	Virus
TBT 235	Texas, USA	1971	Western equine encephalitis
71V-1658	Alberta, Canada	1999	Western equine encephalitis
85-452NM	New Mexico, USA	1985	Western equine encephalitis
AG80-646	Chaco, Argentina	1980	Western equine encephalitis
71V1658	Oregon, USA	1971	Western equine encephalitis
BFS-2005	California, USA	1971	Western equine encephalitis
Montana-64	Montana, USA	1967	Western equine encephalitis
Kern	California, USA	1974	Western equine encephalitis
Imperial	California, USA	2005	Western equine encephalitis
McMillan	Ontario, Canada	1941	Western equine encephalitis
ON41-McMillan	Ontario, Canada	1941	Western equine encephalitis virus
TBT 235 (2)	Texas, USA	1971	Western equine encephalitis virus
CO92-1356	USA	1992	Western equine encephalitis
71V-1658	Alberta, Canada	1999	Western equine encephalitis
585-01	Georgia, USA	2001	Highands J virus
TC-83	Venezuela	1986	Venezuelan equine encephalitis
8131	Peru	2008?	Venezuelan equine encephalitis
71D-1252	Peru	1971	Venezuelan equine encephalitis
MX01-22	Mexico	2004	Venezuelan equine encephalitis
80U76	Unknown	2001?	Venezuelan equine encephalitis
OAX142	Mexico	1996	Venezuelan equine encephalitis
CPA201	Mexico	1993	Venezuelan equine encephalitis
OAX131	Mexico	1996	Venezuelan equine encephalitis
CPA152	Mexico	1996	Venezuelan equine encephalitis
Mena II	Panama	1962	Venezuelan equine encephalitis
68U201	Unknown	1968	Venezuelan equine encephalitis
Cabassou CaAr 508	Guyana	1968	Venezuelan equine encephalitis
Pixuna BeAr 35645	Brazil	1961	Venezuelan equine encephalitis
Mucambo BeAn 8	Brazil	1964	Venezuelan equine encephalitis
78V-3531	Brazil	1968	Venezuelan equine encephalitis
AG80-663	Argentina	1980	Venezuelan equine encephalitis
254934	Venezuela	2000	Venezuelan equine encephalitis
3880	Panamá	1988?	Venezuelan equine encephalitis
V3526	USA	2003?	Venezuelan equine encephalitis
3908	Venezuela	1995	Venezuelan equine encephalitis
6119	Venezuela	1995	Venezuelan equine encephalitis
PMCHo5	Venezuela	1995	Venezuelan equine encephalitis
PE6	USA	2004?	Eastern equine encephalitis
82V2137	USA	1982	Eastern equine encephalitis
NJ/60	USA	Unknown	Eastern equine encephalitis
FL93-939	USA	1993	Eastern equine encephalitis
Florida91-4697	USA	1991	Eastern equine encephalitis
Georgia 97	USA	1997	Eastern equine encephalitis
ssp. North American variant	USA	1991?	Eastern equine encephalitis
PE-3.0815	Peru	1996	Eastern equine encephalitis
YN0540	China	2005	Getah
HB0234	China	2002	Getah
LEIV 17741 MPR	Mongolia	Unknown	Getah
Getah virus, swine	Korea	Unknown	Getah
Mayaro virus	Brazil	Unknown	Mayaro
MAYLC	French Guiana	Unknown	Mayaro
Gulu strain	Africa	Unknown	O´nyong-nyong
SG650	Uganda	1996–1997	O´nyong-nyong
IBH10964	Nigeria	1966	Igbo Ora
DRDE-06	India	2006	Chikungunya
IND-06-TN1	India	2006	Chikungunya
IND-06-MH2	India	2006	Chikungunya
IND06KA3	India	2006	Chikungunya
IND06KA9	India	2006	Chikungunya
IND06KA12	India	2006	Chikungunya
MIDV857	Zimbabwe	1993	Middelburg
Middleburg strain	Unknown	Unknown	Middelburg
Edsbyn 82-5	Sweden	Unknown	Ockelbo
YN87448	China	1998?	Sindbis
S.A.AR86	South Africa	Unknown	Sindbis
HR wt strain	Unknown	Unknown	Sindbis
